# WHEN AND HOW I WANT: FEASIBILITY AND EFFECTS OF EMBEDDING PHYSICAL ACTIVITY INTO NURSING HOME CARE

**DOI:** 10.1093/geroni/igz038.597

**Published:** 2019-11-08

**Authors:** Eva Barrett, Paddy Gillespie, John Newell, Dympna Casey

**Affiliations:** National University of Ireland Galway, Galway, Ireland

## Abstract

Physical activity (PA) is essential to maintaining health into older age. However, older adults living in nursing homes (NHs) remain highly inactive. This study tested the feasibility of a PA programme embedded into NH care and its potential effects on older adults’ function and quality of life (QOL). A cluster-randomised controlled pilot feasibility study, including qualitative and economic components, was conducted. Intervention participants (n=18) performed Morning Movement (morning-time walking and sit-to-stand exercises) and Activity Bursts (bouts of activity throughout the day in standing), 3 times weekly for 12-weeks. Participants in the control NH (n=16) received usual care. At baseline and 12-weeks, feasibility and economic data were collected, function was measured using the Timed Up and Go (TUG) and 10-Metre Walk Test (10MWT) and QOL was measured with the Nottingham Health Profile (NHP) and Investigating Choice Experiments for the Preferences of Older People-CAPability (ICECAP-O). Semi-structured interviews were conducted with staff and participants at mid- and post-intervention and analysed thematically. The PA programme was acceptable to staff and participants and study procedures were feasible. Mean TUG improved by 10.2 (±21.6) seconds in the intervention group and was unchanged -0.2 (9.5) seconds in the control group (95% confidence interval of between-group difference in improvement -2.5 to 23.3 seconds). 10MWT scores stayed stable in the intervention group and disimproved in the control group. ICECAP-O and NHP scores were unchanged. While this study contained a small sample, it demonstrated a feasible, acceptable and potentially effective NH PA intervention and provides guidance for a definitive trial.

